# The role of ontologies in biological and biomedical research: a functional perspective

**DOI:** 10.1093/bib/bbv011

**Published:** 2015-04-10

**Authors:** Robert Hoehndorf, Paul N. Schofield, Georgios V. Gkoutos

**Keywords:** ontology, Semantic Web, data integration, data mining

## Abstract

Ontologies are widely used in biological and biomedical research. Their success lies in their combination of four main features present in almost all ontologies: provision of standard identifiers for classes and relations that represent the phenomena within a domain; provision of a vocabulary for a domain; provision of metadata that describes the intended meaning of the classes and relations in ontologies; and the provision of machine-readable axioms and definitions that enable computational access to some aspects of the meaning of classes and relations. While each of these features enables applications that facilitate data integration, data access and analysis, a great potential lies in the possibility of combining these four features to support integrative analysis and interpretation of multimodal data. Here, we provide a functional perspective on ontologies in biology and biomedicine, focusing on what ontologies can do and describing how they can be used in support of integrative research. We also outline perspectives for using ontologies in data-driven science, in particular their application in structured data mining and machine learning applications.

## Introduction

The past 15 years have seen a revolution in the volume and complexity of data created in the life sciences, and with the increase in available data, the need for data management, integration and analysis has become an increasingly important challenge. The use of ontologies began in the biological sciences around 1998 with the development of the Gene Ontology (GO) [1]. By 2007, there was sufficient interest and activity in the area to merit national and international coordination efforts such as the Open Biomedical Ontologies (OBO) Foundry [2] or the National Center for Biomedical Ontologies [3].

Many definitions of ‘ontology’ have been proposed in the literature [4–10], and classifications of different types of vocabularies, thesauri, ontologies and knowledge bases have been proposed, based on criteria such as their intended use, degree of formalization or philosophical interpretation [2, 11–15]. Independent of the actual definition of what an ‘ontology’ is, most artifacts labeled ‘ontologies’, as well as some ‘vocabularies’ and ‘thesauri’, provide several main features, and these features are used in almost all their applications (see Table 1):

- classes and relations, referred to by an identifier such as an Internationalized Resource Identifier (IRI), a Uniform Resource Identifier (URI), or a database identifier string;
- a domain vocabulary, i.e. a list of terms associated with the ontology’s classes and relations;
- textual definitions and descriptions that provide additional information about what kind of things a class or relation refers to,
- formal definitions and axioms that provide a computational counterpart to textual definitions and that can be accessed and exploited automatically using specialized software (i.e. automated reasoners) and axioms about a domain, i.e. statements that are considered to be true within that domain and which provide background knowledge about a domain.

**Table 1.** The main features provided by ontologies in support of biological and biomedical research

Ontology feature	Utility in research
Classes and relations	The use of standard identifiers for classes and relations in ontologies is what enables data integration across multiple databases because the same identifiers can be used across multiple, disconnected databases, files, or web sites.
Domain vocabulary	Through labels associated with classes and relations, ontologies provide a domain vocabulary that can be exploited for applications ranging from natural language processing, creation of user interfaces, etc.
Metadata and descriptions	Textual definitions, descriptions, examples and further metadata associated with classes in ontologies are what enable domain experts to understand the precise meaning of class in the ontology. The definitions and related metadata should allow consistent understanding of the meaning of classes in ontologies.
Axioms and formal definitions	Formal definitions and axioms enable automated and computational access to (some parts of) the meaning of a class or relation.

Here, we discuss ontologies as artifacts containing these features, and we use these features to provide a ‘functional’ perspective on ontologies (as well as other artifacts such as thesauri, glossaries, semantic networks, or structured vocabularies that provide a similar functionality). We illustrate how these features can be exploited to enable or improve data analysis in biology and biomedicine, and how the combination of these features makes data integration and data analysis across traditional domain boundaries a reality.

## A functional perspective on ontologies

### Classes and relations

The principal components of ontologies are classes and relations. A ‘class’ is an entity that refers to a set of entities in the world, such as the class ‘Protein’ (referring to the set of all proteins), ‘Apoptosis’ (referring to the set of all apoptotic processes) or ‘Red’ (referring to the set of all red qualities). However, in contrast to sets that are defined by their extension (i.e. the entities that are part of the set), classes in ontologies are defined ‘intensionally’ by specifying the properties, features and relations that the entities belonging to a class must have [6, 9]. Relations are similar to classes but hold for two or more entities. Examples are the relations ‘part of’, ‘participates in’ or ‘quality of’.

**Figure 1.**
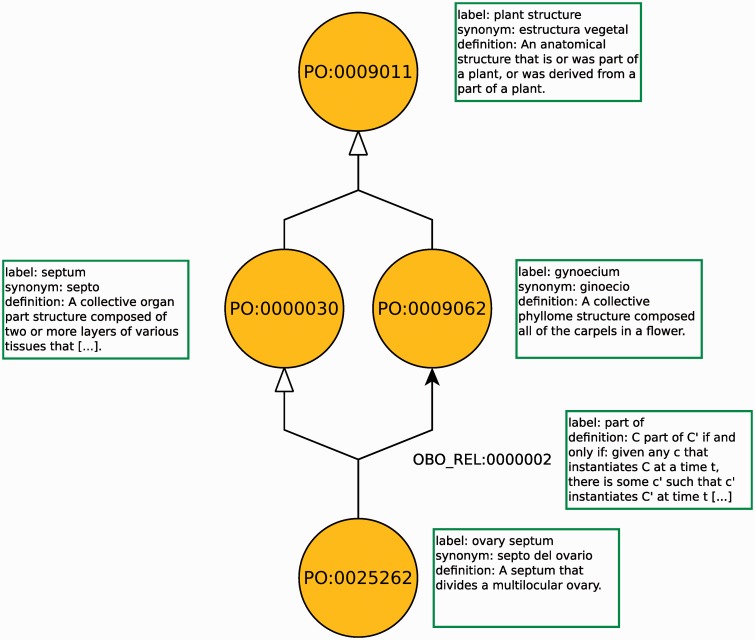
A part of the Plant Ontology. The figure shows classes as circles, labels and definitions in boxes and axioms as edges between classes. The label and definition of the relation OBO_REL:0000002 is a label for an axiom pattern.

In ontologies, classes and relations are commonly referred to using a unique identifier. In the Semantic Web [16], this identifier is an IRI, which is a URI supporting Unicode characters. It is still common to use database identifier strings in biomedical databases to refer to classes and relation. For example, within the OBO [2] community, an identifier for a class or relation in an ontology consists of a prefix string, a colon and a series of digits [17]. In Figure 1, PO:0009011 is an identifier for a class and OBO_REL:0000002 an identifier for a relation, with the prefixes PO and OBO_REL, respectively. In communities in which database identifiers are still widely used, transformation policies that standardize how database identifiers are transformed into IRIs may be adopted. For example, within the OBO, PO:0009011 would be translated to the IRI http://purl.obolibrary.org/obo/PO_0009011 [17].

### Domain vocabulary

The second main feature that ontologies provide is a set of labels associated with the classes and relations in the ontology. Labels are strings that are used to refer to the kind of things a class or relation represents. In ontologies, labels may be provided in multiple languages, and multiple labels may be assigned to one class. Additionally, a primary label may be distinguished from secondary labels or synonyms. Such an assertion signifies that, within the context of an ontology, the primary label is what is used to refer to a class or relation, while the additional labels and synonyms are used to refer to the phenomena captured by a class or a relation in other contexts.

In some ontologies or structured vocabularies, the (primary) label of a class is also used as component of the class identifier (its IRI), but in the majority of ontologies the label and the class identifier are maintained as distinct features, as the label may change (in the simplest case owing to a misspelling) while the intended meaning of the class remains the same [18, 19]. The distinction between label and class identifiers caters for changing metadata associated with the class without having to modify data that are already characterized with the class identifier.

Provision of a domain vocabulary is a widely used feature of ontologies. If an ontology aims to cover a domain completely, the set of labels associated with the ontology classes and relations provide a large set of relevant terms within that domain. For example, an ontology for human anatomy such as the Foundational Model of Anatomy [20] will not only contain the classes and relations relevant to describe human anatomy, but also provide a large set of terms used to refer to human anatomical structures and the ways in which they may be related (as labels of the relations).

### Textual definitions, descriptions and metadata

A third feature of ontologies is the provision of information about the kind of phenomena a class or relation is supposed to capture. The majority of ontologies contain two main kinds of additional information: the first is intended primarily for users of the ontology and provides textual definitions, examples and background information that makes the intended meaning of a class in the ontology as precise as possible to ontology users; the second is additional technical information that relates one class to entries in other databases, literature or other ontologies and vocabularies.

Most ontologies in biomedicine that are primarily intended for data annotation across multiple databases provide textual definitions for their classes. There has been some discussion about what constitutes a ‘good’ textual definition in ontologies [21]. In some domains, ontology users have opted to use Aristotelian definitions, i.e. definitions that state the general kind of thing that a class or relation represents, coupled with the properties that distinguish it from the general kind (the ‘genus–differentia’ model). For example, an ‘ovary septum’ can be defined as a ‘septum’ (the general kind) that ‘divides a multilocular ovary’ (the conditions or properties that separate it from others within the general kind). However, other types of textual definitions are widely used as well [22]. Ideally, the textual definitions are sufficient for an ontology user to understand exactly what kinds of phenomena a class in an ontology refers to, and a ‘good’ definition does exactly that: it is understandable to an ontology user and removes ambiguity in a term so that different ontology users can apply it consistently.

### Formal definitions and axioms

Finally, ontologies provide ‘formal’ and ‘machine-readable’ definitions and axioms. These are some of the most valuable features of ontologies, as these may enable graph- and network-based analyses, ‘fuzzy’ matches in searches, verification of data consistency, as well as provide background knowledge about a domain and reveal new knowledge through deductive inference. The axioms and definitions of ontologies can be represented in many forms. In some cases, they are expressed directly as a graph structure that is intended to represent a taxonomy or a partonomy. In other cases, axioms and definitions are written in a formal language. For example, Figure 1 represents a part of an ontology as a graph in which the edges ending with a white triangle represent ‘taxonomic’ relationships and the edge labeled ‘part of’ represents a parthood relationship.

Ontologies are increasingly being expressed directly in a formal language, and graph representations of ontologies are being derived dynamically from this formal representation. Most commonly, ontologies in the biological and biomedical domain are represented in the Web Ontology Language (OWL) [23], a formal language based on description logics [24, 25], or a sub-language or profile of OWL such as OWL-EL [26]. The graph-based OBO Flatfile Format, which is still used by several ontologies (currently, in November 2014, 66 ontologies in the OBO library are represented natively in the OBO Flatfile Format, while 45 are represented natively in OWL), has now become a sub-language of OWL [27] and can be processed with the same tools and libraries used for OWL ontologies. Table 2 provides an overview over different representation and query languages for ontologies as well as key concepts centered around ontologies.

**Table 2.** Query and representation languages, and key concepts around ontologies in biology

Language	Description
Resource Description Framework (RDF)	RDF [120] is a graph-based language in which resources are identified through their IRI and statements take the form of triples (subject–predicate–object). Therefore, a set of RDF statements forms a labeled directed graph. RDF also comes with a predefined vocabulary that can be used to state the type of a resource (e.g. a class, or a literal) or represent relations between resources (e.g. labels of resources, subclass relations between resources).
Web Ontology Language (OWL)	OWL [23] is a language based on description logic and has a formal, model-theoretic semantics. Several sub-languages of OWL have been developed, including OWL-DL, OWL-EL, OWL-RL, OWL-QL and OWL Full, which support different language constructs, have different properties regarding decidability and complexity of reasoning tasks, and therefore different areas of application.
SPARQL Protocol and RDF Query Language (SPARQL)	SPARQL [121] is a standardized NoSQL query language, which can be used to query RDF databases and supports query federation (i.e. querying data distributed across multiple databases). SPARQL can also be used to query other kinds of data, including relational databases and flat files.
Linked Data	Linked Data [122] represents a method of publishing and sharing data on the web. When publishing Linked Data sets, data items are identified through a URI, and links to other data items are included in the data set by explicitly referring to the URI that denotes the other items. The URIs used to denote data items should be dereferencable, i.e. it should be possible to obtain additional information about the item through the URI (depending on the method used to access the URI, the information could be presented as HTML, RDF, JavaScript Object Notation or similar).
OBO Flatfile Format	The OBO Flatfile Format [27] is a graph-based knowledge representation language widely used for biological and biomedical ontologies. The majority of language constructs are compatible with OWL, and bi-directional transformations between the OBO Flatfile Format and OWL have been implemented.
Proprietary graph-based ontology representation formats	A number of graph-based representations of ontologies have been developed that primarily specify labeled graphs. Examples include the representation of the Medical Subject Headings thesaurus [123], the Unified Medical Language System [124] or the medical vocabulary SNOMED CT [125].

The construction of ontologies in a formal language often follows—explicitly or implicitly—the axiomatic method [28]. According to the axiomatic method, knowledge about a domain is formalized by first introducing a set of terms referring to classes and relations in the domain (the classes and relations of the ontology), and then explicitly defining these classes and relations by reference to other terms or relations, and possibly introducing new terms and relations. For example, the class ‘ovary septum’ (PO:0025262) in Figure 1 could be defined using the OWL language as:

’ovary septum’ equivalentTo: septum and divides some ’multilocular ovary’

This definition states that the class on the left of equivalentTo: (i.e. ‘ovary septum’) is equivalent to the expression on the right of equivalentTo: (septum and divides some ’multilocular ovary’), making ‘ovary septum’ a shorthand form of the complex statement on the right (i.e. every occurrence of ‘ovary septum’ could be replaced with the expression on the right). A definition alone does not add any information about the intended meaning of a class: the meaning of ‘ovary septum’ now depends entirely on the meaning of ‘septum’, ‘multilocular ovary’ and the relation ‘divides’. Following the axiomatic method, we can introduce further definitions for some of these terms. For example, ‘multilocular ovary’ could be further defined:

’multilocular ovary’ equivalentTo: ovary and has-quality some multilocular

Similarly, since this takes the form of an explicit definition (through the use of the equivalentTo: keyword), we can now replace every occurrence of ‘multilocular ovary’ with the expression on the righthand side. Applying this property of explicit definitions, we can rewrite the definition of ‘ovary septum’ as:

’ovary septum’ equivalentTo: septum and divides some

 (ovary and has-quality some multilocular)

Now, the meaning of the class ‘ovary septum’ depends on the meaning of the classes ‘septum’, ‘ovary’, ‘multilocular’, as well as the relations ‘divides’ and ‘has-quality’. We could continue defining these classes by introducing additional classes and relations. However, inevitably, we will come up with a set of classes and relations that we cannot further define.

As a second step in the axiomatic method, we use the ontology’s classes and relations in statements that we consider to be true in the domain it is supposed to represent. These statements are the axioms, which form the features of ontologies that provide domain knowledge and fill the classes and relations with meaning. For example, we could state about the ‘has quality’ relation that, if an entity x has the quality q, and an entity y has the quality q, then x must be identical to y (i.e. a quality is always the quality of at most one entity). In OWL, we could state this simply as:

ObjectProperty: ’has quality’

Characteristics: InverseFunctional

Another kind of axiom is the ‘subClassOf:’ axiom in which one class is asserted to be a subclass of another class. A class X is a subclass of Y if and only if all instances of X are also instances of Y (i.e. all things satisfying the conditions for X also satisfy the conditions for Y). In Figure 1, these axioms are illustrated as arrows with white triangular pointers. Subclass axioms do not always take the form of simple assertions of a subclass relation between two named classes, but may involve more complex class expressions as well. For example, the ‘part of’ axiom in Figure 1 would be expressed in OWL as:

’ovary septum’ subClassOf: ’part of’ some gynoecium

Here, ‘ovary septum’ is a named class in the ontology while ’part of’ some gynoecium is a complex class expression involving the relation ‘part of’, the named class ‘gynoecium’ and the existential quantifier some.

Ontologies that are formalized in OWL may contain many more kinds of axioms [29], and some ontologies that are formalized in more expressive languages than OWL, such as first- and second-order predicate logic [30], may contain a large variety of axioms. Examples of ontologies that are formalized at least in parts in such expressive languages include the RNA Ontology [31], the Basic Formal Ontology [32] or parts of the Sequence Ontology [33, 34].

The axioms and definitions in ontologies can give rise to a graph structure that can be exploited using graph- and network-based algorithms. In these graphs, nodes commonly represent classes, and edges represent types of axioms that hold between these classes [35]. In particular, ontologies give rise to ‘taxonomic graphs’, which represent the subclass relations between the named classes in the ontology. Another pattern that is frequently used in generating a graph structure from ontology axioms is the existential restrictions on the ‘part of’ relation to give rise to a partonomy [36]. Here, an edge labeled ‘part of’ is generated between classes X and Y if X is a subclass of ’part of’ some Y. Importantly, the label of the edge between classes (e.g. ‘part of’) is different from the relation ‘part of’ that holds between the instances of the class [37]; the label of the edge is a shortcut for the complex axiom pattern involving the two classes (or a relation between the two classes that is explicitly defined using such an axiom pattern).

## Using ontologies

Several tools and methods have been developed that make use of ontologies and support their use. These tools often focus on one or two of the features of ontologies, and here we distinguish them by the main task they aim to support.

### Annotation and data integration

The use of standard identifiers for classes and relations in ontologies is a key component in enabling data integration across multiple databases, because the same identifiers can be used across multiple, disconnected databases, files or web sites. Consequently, these identifiers are widely used in structured file formats, in knowledge bases and data repositories. In fact, one of the first applications for which biological ontologies were developed, notably the GO [1], was to make biological sense of the large data sets emerging from the new expression array technologies in the early 2000s. Differential expression screens and Serial Analyis of Gene Expression (SAGE) analyses generated data sets of often thousands of genes, which needed to be interpreted in terms of gene function. This provided the impetus behind the ongoing functional and structural annotation of gene products, which is now available through the GO database [38] and is a mainstay of modern bioinformatics. In particular, ontologies enabled the assignation of functions to gene products and the ability to compare these functions computationally within and across species; these features have become key tools in functional and comparative genomics.

At its core, an ontology-based annotation associates an entity and an ontology class, and combines this assertion with metadata that contains, among others, information about who created the annotation, the date at which the annotation was created or the evidence that was considered. The entity that is annotated can be represented by an identifier in a database, referred to by a word or phrase in text, or even visually represented in an image [39, 40]. Annotation tools are concerned with recording the annotation in standard formats, performing basic quality checks and providing the metadata for the annotations, as well as suggesting or inferring ontology-based annotations using custom algorithms. For example, when the annotations refer to entities mentioned in text, annotation tools may use natural language processing techniques, such as named entity recognition and relation extraction, and when annotations refer to entities represented in images, image processing techniques may be applied.

The majority of annotation tools allow for the inclusion of provenance information, such as the evidence for an ontology-based annotation as recorded using the Evidence Code Ontology [41] or the Provenance Ontology [42]. Tools such as Domeo [43], an annotation framework applied among others by the Neuroscience Information Framework and the OpenPhacts projects, uses the Annotation Ontology [39] to formally capture provenance information associated with ontology-based annotations. Furthermore, an increasing number of annotation tools use the W3C Open Annotation Data Model [44], or are able to import and export annotations in this format.

Annotation tools that support curators through markup of literature are widely used to suggest possible annotations [45]. For example, the Textpresso software tool [46] was one of the first tools developed to support literature curation for GO, and is still extensively used in model organism databases [47]. Some annotation tools come with additional functionality to allow interactions between curators of data sets and ontology developers. For example, the Phenex tool was designed to support the phenotype annotation of character matrices in the Phenoscape project [48]. Phenex contains workflow elements and inbuilt reliability algorithms that aim to reduce curator workload [49]. Furthermore, Phenex also allows feedback to ontology developers to request new ontology classes that are needed to capture data accurately. While Phenex is primarily an annotation tool relying on input from literature and experts, other tools can incorporate domain-specific algorithms to aid in the annotation process. For example, the GO consortium [38] applies the Phylogenetic Annotation and Inference Tool, which assists curators to infer annotations among members of a protein family based on sequence orthology [50], making GO an interesting example of the confluence of the use of manual assignment based on published evidence, and electronic inference (by orthology or structural motif) to fill the gaps in our knowledge concerning gene product function and location.

Data integration and annotation go hand-in-hand, and in particular for complex multimodal data sets, annotation with single ontologies is often not sufficient. A particularly complex use-case of annotation with multiple ontologies occurs in the domain of phenotype descriptions, as applied in large-scale mutagenesis projects. For example, in the Zebrafish Mutagenesis Project [51], much of the observed data is categorical and describes anatomical and physiological variation, and the phenotypic descriptions are based on anatomy and process ontologies [51]. The International Mouse Phenotyping Consortium (IMPC) [52], on the other hand, generates both categorical data, which are assigned by investigators directly based on a phenotype ontology, and quantitative data. The strategy adopted by the IMPC is to express phenodeviance by assigning a class from a phenotype ontology on the basis of predetermined statistical thresholds [53, 54]. This form of automated annotation, albeit on highly quality-controlled data, is time-efficient and facilitates data integration and mining across qualitative and quantitative information.

When it becomes necessary to use more than a single ontology for annotation, it is beneficial to fix the ontologies that are being used to annotate a data set. Ontology repositories (Table 3) can aid in finding ontologies suitable for annotating data within a domain.

**Table 3.** Overview of main ontology repositories in the life science domain

Repository	Key features	URL
BioPortal	BioPortal [126] is the largest ontology repository for ontologies in biology and biomedicine. It contains >400 ontologies with a total of >6 million classes. BioPortal can be used to find ontologies based on the ontology name or the label of a class within the ontology. It further has a large number of web services and widgets that allow embedding of key BioPortal functions in web applications. The NCBO Annotator [127] is a part of BioPortal and can be used to find labels of ontology classes in text. BioPortal can also be accessed through a SPARQL endpoint.	http://bioportal.bioontology.org/
OntoBee	Ontobee [128] is an ontology repository in which ontologies are presented as Linked Data. Ontobee provides information about the classes and relations used by the OBO project.	http://www.ontobee.org/
Ontology Lookup Service	The Ontology Lookup Service [129] consists of a repository of ontologies represented in the OBO Flatfile Format, and enables search of single ontologies, lookup of terms across multiple ontologies and browsing and visualizing the ontology graph structures. The Ontology Lookup Service can be accessed through a web interface and a number of web services.	http://www.ebi.ac.uk/ontology-lookup/
OBO Library	The Open Biological and Biomedical Ontologies (OBO) library [2] consists of a number of ontologies that have been developed according to a set of agreed principles including complementarity and collaborative development.	http://obofoundry.org

### Ontologies as vocabularies

Ontologies provide vocabularies of the terms used within a domain. Therefore, they can be used by a large variety of applications that rely on domain-specific terms. Example applications for the vocabulary component of ontologies include user interfaces for databases that contain ontology-based annotations, and natural language processing methods.

Tools using the vocabularies associated with ontologies use them in two main ways. First, the labels of an ontology classes and relations enable access to data or text annotated with these ontologies. For this type of application, a link is established between a class and a user-readable name of that class. This link is then used to provide a way for human users of an ontology to access the information associated with the ontology class. Tools that use this feature include a wide range of browsers that enable access to ontology-based annotations through the class labels, such as the Amigo tool [55], which enables access to GO annotations, or GOPubMed [56], which enables access to scientific articles based on ontology classifications.

Second, the labels in an ontology can be used to identify whether the text mentions a phenomenon characterized by a class or relation in an ontology. Applications of this type typically require the utilization of natural language processing techniques [57]. One example of such application is the NCBO Annotator, a tool that can recognize the labels and synonyms of ontology classes in natural language texts [58]. The National Center for Biomedical Ontologies (NCBO) Annotator implements a basic concept recognition approach [59] that generalized well across multiple vocabularies and does not require additional training. However, more specialized approaches have been developed, in particular in the context of recognizing descriptions of gene functions and biological processes in text [60], which can then be used to develop software tools that assist domain experts in literature-based database curation.

The labels of classes in ontologies can also be used for large-scale text mining to identify system-wide associations between the phenomena to which they refer. Text mining based on ontologies has been used to identify the presence of disease modules based on phenotypes [61, 62], drug targets and drug indications [63, 74], drug–drug interaction [65] and candidate genes for diseases [66, 67]. The success of these methods depends on the coverage of terms used to refer to classes in the ontology.

The main challenge in relying on class labels to recognize the reference to an ontology class in text is that labels do not capture all of the possible linguistic variations around terms and phrases used to refer to an ontology class [68]. Recognizing ontology classes referenced in text poses a distinct set of challenges, in particular for semantically complex classes, or classes for which no common and widely used terms have been established [69–71].

### Formalized definitions and axioms: reasoning with ontologies

Several tools and software libraries can make use of ontologies’ axioms and formal definitions. The primary means to access and process ontologies semantically are automated reasoners, i.e. software tools that can directly infer knowledge from the axioms and definitions in ontologies using deductive inference. Automated reasoners can detect contradictions in the axioms and definitions of an ontology (consistency checking), infer the most specific subclasses and superclasses for all classes in an ontology (classification) and answer complex queries. A wide range of automated reasoners has been developed for different subsets of OWL, supporting different features and exhibiting different computational complexity for basic reasoning tasks such as answering queries (Table 4). Reasoners for subsets of OWL such as OWL-EL support less expressivity for axioms and queries in ontologies, but usually guarantee a lower computational complexity. For complex ontologies expressed in OWL, examples of commonly used reasoners include Pellet [72] owing to its support for a large number of features, and HermiT [73] owing to its high performance for complex ontologies. For ontologies expressed in the OWL-EL profile, the ELK reasoner [74] is widely used owing to its support for large ontologies and parallel reasoning. Recent developments include the Konklude reasoner [75], which outperforms most OWL-EL and OWL 2 reasoners even for large ontologies [76]. As reasoner technology is evolving rapidly, new optimization methods can lead to significant performance improvements. If a selected reasoner cannot perform a reasoning task over an ontology, it can pay off to review reasoner competitions such as the annual OWL Reasoner Evaluation workshops [76] to find another reasoner that is more adequate for an ontology and desired application. Alternatively, ontology modularization approaches [23, 77–79] can be applied to extract subsets of ontologies, which automated reasoners can process efficiently.

**Table 4.** A selection of automated reasoners for OWL ontologies

Reasoner	OWL support	Description
Pellet [72]	OWL 2, OWL EL	General purpose OWL reasoner with a large set of features, including specialized OWL EL reasoning, support for rules, support of epistemic operators, integration in SPARQL, explanation of inferences, incremental reasoning.
HermiT [73]	OWL 2, OWL EL	General purpose, highly optimized OWL reasoner.
FacT++ [130]	OWL-DL, OWL 2 (partially)	Highly optimized reasoner implemented in C++.
Konklude [75]	OWL 2	Highly optimized OWL reasoner supporting parallel reasoning.
RacerPro 2.0 [131]	OWL 2 (partial)	Optimized OWL reasoner, with integration in the AllegroGraph [132] triple store.
TrOWL [133]	OWL 2	Scalable OWL reasoner with support for limited closed-world reasoning (negation as failure) and stream reasoning.
ELK [74]	OWL-EL	Optimized and feature-rich OWL EL reasoner with support for incremental and parallel reasoning.

OWL reasoners are either implemented as stand-alone tools, or can be accessed through the OWL API [80] or the OWLLink protocol [81]. The OWL API is a reference implementation for creating and manipulating OWL ontologies and provides interfaces for automated reasoning that the majority of OWL reasoners implement. OWLLink is an HTTP-based protocol for communicating with OWL reasoners. Reasoners can also be accessed through ontology editors such as Protege [82]. Table 5 provides an overview of some common tools and software libraries used to process ontologies and interact with reasoners, and Table 6 shows some common analysis and visualization tools and libraries that use ontologies.

**Table 5.** An overview over tools and software libraries for processing and interacting with ontologies

Tool	Description	Web site
Protege, WebProtege	Protege [82] is an OWL ontology editor with full support for OWL ontologies and a large number of plug-ins that provide integration of reasoners, export and import of various ontology representation formats, or ontology visualization. WebProtege is a web-based collaborative ontology editor, which provides similar functionality to Protege through a web interface.	http://protege.stanford.edu/, http://webprotege.stanford.edu/
OWL API	The OWL API [80] is a reference implementation and a *de facto* standard for processing OWL ontologies.	http://owlapi.sourceforge.net/
Owlcpp	owlcpp [134] is a C++ library for processing OWL ontologies. It includes support for querying ontologies through automated reasoners.	http://owl-cpp.sourceforge.net/
Brain	Brain [135] is a library based on the OWL API that provides convenience methods for processing and reasoning with ontologies, in particular biological and biomedical ontologies represented in the OWL-EL profile of OWL.	https://github.com/loopasam/Brain
Redland RDF API	An RDF library written in C. It provides a large set of commonly used command line tools to transform or collect basic statistics about an RDF file.	http://librdf.org/
Apache Jena	Jena is a Java library and collection of tools consisting of an RDF library, integration of SPARQL queries and support for OWL ontologies.	https://jena.apache.org/

**Table 6.** An overview over generic ontology analysis and visualization tools and libraries

Tool	Description	Web site
Gephi	Gephi [136] is a generic graph-visualization tool, and can be used to visualize classes and relations in ontologies. Gephi also supports a number of algorithms for basic graph analysis, including transitive inference over edges.	http://gephi.github.io/
Cytoscape	Cytoscape [137] is a tool for visualizing and analyzing interaction networks and other graphs including ontologies. Several Cytoscape plug-ins support using ontologies for visualization and analysis.	http://www.cytoscape.org/
Semantic Measures Library	The Semantic Measures Library and Toolkit [138] is a generic framework implementing a large variety of semantic similarity measures over ontologies.	http://www.semantic-measures-library.org/
GO enrichment analysis tools	Enrichment analysis uses the graph-structure underlying ontologies (usually the GO) together with transitive inference over the edges in the graph to statistically test a hypothesis. The graph structure is used to ‘enrich’ statistical power by propagating annotations transitively over the graph and performing a test at each level of the ontology hierarchy.	http://geneontology.org/page/go-enrichment-analysis
OntoFUNC	OntoFUNC [139] is a software tool to perform ontology enrichment analysis over arbitrary OWL ontologies.	http://phenomebrowser.net/ontofunc/

Most users of ontologies will not access ontologies directly through automated reasoners, but will either use the output of an automated reasoner (e.g. the inferred graph structure of an ontology) or interact with a reasoner indirectly (e.g. through a software tool that uses an automated reasoner as part of its operation). Nevertheless, in some approaches, automated reasoning has been applied directly to verify data consistency with respect to constraints in an ontology or reveal novel biological knowledge based on axioms in an ontology. The axioms in an ontology can be used to verify whether an entity described in a database is able to satisfy the conditions laid out for that kind of entity, and automated reasoning can be used to detect conflicts. For example, such an approach has been applied retrospectively to computational models in systems biology [83], but is increasingly being applied to ontology-based annotations at the time the annotation is made [84, 85]. Some data exchange standards are now being designed with data verification in mind, and a prime example is the BioPAX standard for pathway data sharing, which is based on formalized knowledge in OWL [86]. The axioms in an ontology can also be used to infer the class to which an entity belongs based on the features and descriptions of the class and the entity. An application of this is the inference of the protein family to which a protein belongs based on an ontology and automated reasoning [87].

More subtly, reasoning over ontologies can also be applied for integrating ontology-annotated data sets across different domain by systematically combining different ontologies using axioms or axiom patterns [88, 89]. In such applications, the relationship between classes in different ontologies is identified and expressed in the form of an axiom or axiom pattern that is systematically applied to several pairs of classes. Prime examples of this form of integration are species-specific anatomy and phenotype ontologies [90, 91]. Integrating data annotated with these ontologies relies on identifying homologous anatomical structures [92] and relating the classes that refer to these structures in different anatomy ontologies using axiom patterns [90, 93].

### Mining and analyzing multimodal data with ontologies

The great potential in using ontologies for data analysis lies with the possibility of combining their different functional levels, and some exciting insights into the biological properties of whole systems have been achieved by combining data through ontologies. For example, one of the most widely used applications for ontologies is Gene Set Enrichment Analysis [94] or similar enrichment methods, which combine the graph structure of ontologies (axioms and definitions) with their potential for data integration (through ontology-based annotations) to provide a statistical interpretation of differences between two states with regard to the background knowledge provided by the ontology over which the enrichment analysis was performed. Another analysis method specifically relying on ontologies and their annotations is the use of similarity measures to determine the ‘semantic’ distance and proximity between data items [95]. In semantic similarity measures, the axioms and definitions of ontologies are exploited to define a similarity between annotated data items. Semantic similarity has widely been applied to computationally predict protein–protein interactions based on their functional similarity [96, 97], to the diagnosis of disease based on phenotypic similarity [98–100], or to the classification of chemicals based on structural similarity [101].

While statistical analysis of graphs or sets, or measures of semantic similarity, are well established methods that use ontologies for data mining, many machine learning and data mining algorithms that are applied to unstructured data are not yet widely used with ontologies and ontology-structured data. The challenges of using these methods occur both when using ontologies and ontology-annotated data as the target of a machine learning and data mining algorithm as well as when using ontologies and ontology-annotated data as features. When using ontologies as the target, i.e. when aiming to learn an ontology-based classification for some piece of data such as the functions of a protein, several challenges arise in relation to the adoption of these traditional algorithms to ontology-based data in the biological and biomedical domains. These challenges primarily relate to the ‘multi-class’ nature of the problem, as ontologies have often very large numbers of classes, the ‘structured dependency relations’ between these classes (i.e. the axioms in the ontology) and, in many cases, the ‘multi-label’ nature of the classification problem as data items are usually annotated to more than one ontology class. When using ontologies, or ontology-annotated data, as features in a machine learning task, challenges relate to the large number of classes that are often sparsely populated (more specific classes are usually present less frequently while more general classes are used more frequently), and again the dependency relations between classes (e.g. disjointness, subclass relations and axiom patterns that exist between classes).

Despite these challenges, progress is being made in incorporating ontologies and ontology-annotated data into machine learning and data mining algorithms. For example, in the area of prediction of protein functions, driven by the Critical Assessment of Function Annotation challenge [102], several approaches have been developed to predict GO annotations of proteins [103–106], some of which use ontologies as features as well [104, 107]. While these methods have been developed in the context of protein function prediction, parts of these can be transferred to other problems.

The use of ontologies can also help address a challenge that machine learning and data mining approaches face: the incorporation of different types of features for multimodal learning and classification [108]. Combining information from text, images, videos, molecular data or structured data in knowledge bases to improve classification can be facilitated through the use ontologies, by first extracting relevant features from each type of information and representing the results using a single ontology that combines the information used for training a classifier.

## Perspective

There are now sufficient stable ontologies to permit routine reuse of classes from multiple ontologies in automated or semiautomated ontology construction algorithms [109]. With increasing size and number of ontologies, the ability to modularize ontologies to generate application-specific ‘views’ while maintaining interoperability with data sets in a domain that are annotated with another module of the same ontology will become essential. A recent example of this is provided by the Bioassay ontology [110] or the automated generation of phenotype ontologies [111, 112]. To support these applications, coverage and quality of content in established ontologies must be further improved [113], a task that poses a serious challenge and requires the sustained engagement of domain experts.

One major application of exploiting multiple ontologies is to formalize the large, unstructured, multimodal and often distributed data from clinical records. It is now possible to capture information and knowledge related to diagnostic procedures, drugs, phenotypes, diseases and genotypes using existing ontologies, and there are efforts to create ontologies for capturing other environmental and behavioral data for patients. Such ontologies are now being applied in a clinical setting [114], but mainly for data mining from partially structured and legacy clinical records [115]. Incorporating ontologies directly in the electronic health record will lead to novel methods for patient classification and stratification, and the analysis and mining of large-scale patient data. With increasing numbers of whole exome and genome sequences in clinics, there is marked potential for using ontology-based enrichment algorithms or incorporating results from basic biological research into clinical decision making [116]. We expect to see further rapid developments in this important area.

From an algorithmic and methodological point of view, the next challenge we face is the development of new methods for applying ontologies in data mining and data analysis. These methods must be able to use the different features ontologies provide, combine them in meaningful ways and be applicable to large, complex and multimodal data sets. We also expect to see more complex ontology-based applications that combine the main features of ontologies in novel ways. For example, annotation tools will be developed that do not merely use the labels and class identifiers to associate entities with ontology classes, but use the ontology’s axioms and formal definitions to preselect possible annotations (e.g. by eliminating possible process classes at places at which only annotations to material objects would be sensible), verify the consistency of an annotation [83], reveal the consequences of asserted information to users [87] and be applicable to multiple types of data (e.g. structured data, text and images).

Finally, to further improve ontology-based data integration and analysis, robust evaluation criteria need to be developed that are based on how ontologies are actually being used in research applications [117]. Recently, some exciting results have demonstrated that the GO accurately resembles modules found in experimentally derived gene and protein interaction networks, leading to a data-driven way for validating an ontology [118]. The increasing use of ontologies in scientific research will lead to improved methods for evaluating ontology quality based on their performance in scientific applications [117, 119]. A tighter integration between experimental results and the domain knowledge formalized in ontologies will not only lead to improved evaluation criteria and subsequently better ontologies, but is also a crucial step in making sense of large structured and unstructured data sets in biology and biomedicine.

**Key Points**

- Ontologies provide identifiers for classes and relations that represent phenomena within a domain, thereby enabling integration of data.
- Ontologies provide labels for classes and relations, thereby providing a domain vocabulary.
- Ontologies provide metadata associated with classes and relations that allows human users to understand their meaning and contribute to consistent use in annotation and other applications.
- Ontologies provide axioms and formal definitions that enable computational access to some aspects of the meaning of classes and relations.
- Combining the four main features of ontologies facilitates semantic integration of heterogeneous, multimodal data within and across domains, and enables novel data mining methods that span traditional boundaries between domains and data types.

## Funding

This work has not received any dedicated funding.
